# Tertiary Lymphoid Structures as a Predictive Biomarker of Response to Cancer Immunotherapies

**DOI:** 10.3389/fimmu.2021.674565

**Published:** 2021-05-12

**Authors:** Marta Trüb, Alfred Zippelius

**Affiliations:** ^1^ Laboratory of Cancer Immunology, Department of Biomedicine, University of Basel, University Hospital Basel, Basel, Switzerland; ^2^ Medical Oncology, University Hospital Basel, Basel, Switzerland

**Keywords:** TLS, tertiary lymphoid structures, tumour, cancer immunotherapy, novel therapies, B cells

## Abstract

Tertiary lymphoid structures (TLS) are ectopic lymphoid formations which are formed under long-lasting inflammatory conditions, including tumours. TLS are composed predominantly of B cells, T cells and dendritic cells, and display various levels of organisation, from locally concentrated aggregates of immune cells, through clearly defined B cell follicles to mature follicles containing germinal centres. Their presence has been strongly associated with improved survival and clinical outcome upon cancer immunotherapies for patients with solid tumours, indicating potential for TLS to be used as a prognostic and predictive factor. Although signals involved in TLS generation and main cellular components of TLS have been extensively characterised, the exact mechanism by which TLS contribute to the anti-tumour response remain unclear. Here, we summarise the most recent development in our understanding of their role in cancer and in particular in the response to cancer immunotherapy. Deciphering the relationship between B cells and T cells found in TLS is a highly exciting field of investigation, with the potential to lead to novel, B-cell focused immunotherapies.

## B Cells in the Cancer Immunotherapy Spotlight

Immunotherapy is a recent breakthrough in oncology treatment which focuses on harnessing the power of the immune system to fight cancer. Immune checkpoint blockade (ICB) therapy mainly targets PD-(L)-1 and CTLA-4 receptors and provides durable responses in cancer patients. T cells have been at the forefront of research surrounding ICB but other immune cells are also increasingly being found to take part in the response (1, 2). Recent studies from human patients (and also mouse models) have put the spotlight on B cells as additional important players in immunotherapy. For example, the presence of B cells and TLS is associated with favourable response to immune checkpoint blockade in patients with soft tissue sarcomas (3), metastatic melanoma (4, 5) and renal cell carcinoma (5). While the field has been so far primarily focused on T cells, these findings call for further investigation into the active role of TLS and B cells in ICB treatment. This review aims to summarise our understanding of the role of B cells and TLS in immunotherapy response and mechanisms in the cellular network of the tumour microenvironment with a focus on the potential cross-talk between B cells and cytotoxic T cells.

## TLS Composition

Orchestrated immune response to cancer is elicited systemically in secondary lymphoid organs (SLO, such as lymph nodes and spleen) and locally, in ectopic lymphoid formations called tertiary lymphoid structures (TLS) found at the tumour site. TLS are composed predominantly of aggregates of B cells and T cells displaying various stages of organisation. Immature TLS present clearly visible foci of immune cells with segregated B and T cell zones, but lack follicular dendritic cells (FDCs) and germinal centres (GCs), with the latter being sites of active B cell proliferation and affinity maturation (6). In the intermediate maturation stage, B and T cell areas are enriched by FDCs but not GCs. Finally, mature TLS contain both FDCs and GCs (7). In general, mature TLS resemble well structure of SLO (8). Of note, TLS are also known to be formed and play role in other chronic inflammatory conditions, such as viral infections (9–11), autoimmune disorders (11–13) and after tissue transplantation (14, 15). Alongside B cells, other immune cells found in the TLS include dendritic cells [DCs (16–18)], CD4+ T follicular helper cells [Tfh (19)], CD4+ regulatory T cells [Tregs (20)], CD8+ cytotoxic T cells (21–23) and macrophages (16), as well as innate lymphoid cells (24). Importantly, TLS are also accompanied by lymphatic and blood vessels (including high endothelial venules), which aid in immune cell trafficking into the tumour (25). Therefore, TLS create the niche which provides opportunity for immune cell interaction in the inflammatory tumour environment.

## The Prognostic Value of TLS in Cancer

The prognostic potential of TLS structures was described for many tumours, including non-small cell lung cancer [NSCLC (26)], colorectal cancer (18, 27), breast (19, 28), pancreatic and gastric cancers (29, 30), melanoma (17) as well as ovarian (31) and oral cancer (32). The presence of TLS carries therefore a positive prognostic value in most solid tumours (33, 34). It is important to keep in mind that different studies used varying methods for TLS quantification, such as the presence of CD208+ DCs found exclusively in TLS (in lung cancer), presence of FDC markers CD21 and CD23 or co-localisation of CD3+ T cells and CD20+ B cells. TLS can be nowadays investigated by state-of the art digital and computational pathology utilizing methods incorporating deep-learning and artificial intelligence (35). Additionally, it is vital to consider other patient-related factors while assessing TLS presence, since co-morbidities (such as chronic inflammation) or treatments (e.g. with corticosteroids) impact TLS formation and maturation (7). Importantly, TLS presence was shown to be independent of tumour mutational burden (which influences immune response to tumours) in several tumour entities (4, 22).

B cells found in tumours display wide variety of phenotypes, ranging from naïve B cells, through actively proliferating GC B cells to memory B cells and terminally differentiated plasma cells. It is important to distinguish between investigations assessing the prognostic role of TLS or individual B cell subsets. For example, presence of memory B cells was associated with poorer survival prognosis in pan-cancer analysis of many solid tumours [including lung squamous cell carcinoma, colon and gastric cancers (36)], although these tumour entities show improved prognosis upon TLS assessment (7, 27). Additionally, in pancreatic cancer, high density of B cells was associated with improved survival prognosis but only if the cells were forming TLS (30). Therefore, it is important to characterise B cell organisation while assessing their prognostic potential. Of note, considering the presence of B cells and TLS often strengthens the prognostic potential of CD8 T cells (4, 37, 38). In ovarian cancer patients, CD8 intratumoral T cells only carried prognostic value in the presence of CD20+ B cells and plasma cells, with the latter population associated with the most robust responses (22).

In conclusion, presence of B cells and TLS is a strong prognostic factor for cancer patient survival on its own and in combination with CD8 T cells, which may suggest an active cooperation of these cell subsets in eliciting successful anti-tumour immune response.

## B Cells in Tumours: Heterogeneity of Phenotypes Leads to Pleiotropic Function

The heterogeneity of B cell phenotypes has functional consequences, as B cell subsets display pleiotropic character. B cell functions fall into two broad categories, namely humoral and non-humoral responses. The humoral responses are consequences of GC reaction and extrafollicular plasma cell activity within TLS and have been extensively reviewed elsewhere (39). Meta-analysis showed positive association of plasma cell signature in most of solid tumours [except for the brain and large cell carcinoma (39)]. Antigen specificity of intratumoral B cells is an emerging topic of interest. Alongside B cells specific for tumour antigens [such as e.g. aberrantly glycosylated mucin 1 (40)], recent studies in patients with head and neck cancer infected with human papilloma virus (HPV) provided evidence of HPV-specific antibody production at the tumour site (41, 42). Whether B cells specific for tumour-derived antigen, self-peptides or viral proteins display different phenotypical and functional state remains to be established.

The non-humoral activities of B cells encompass functions which require direct cell to cell contact, such as antigen presentation (*via* MHC class II and class I molecules) and engagement of co-stimulatory molecules (such as CD40, CD80, CD86, ICOS-L, CD27 and 4-1BBL) or co-inhibitory receptors (including PD-L1 and PD-L2) to CD4 and CD8 T cells. Additionally, B cells secrete a wide range of cytokines, which have potential to influence multiple cell types, including T cells, NK cells and myeloid cells. This includes, again, cytokines with anti-tumoral effects [such as IL-6, IL-12, IL-13, TNF-α and IFN-γ (43)] as well as pro-tumoral character [such as IL-10, IL-35 and TGF-β (44)]. Regulatory B cell subsets (Bregs) which play a tumour-promoting role have also been identified in tumours (45, 46).

Summing up, intratumoral B cells are a multifaceted subset and even though they can display both pro- and anti-tumoral roles (43), there is overwhelming evidence of improved prognosis for cancer patients when B cells form TLS (34). A major challenge is to link B cell phenotypes to effector functions, so that precise therapies aiming at depleting or promoting certain populations can be developed. Additionally, B cell plasticity between different subsets and intra-subset heterogeneity are not fully understood. Whether function of individual cells changes over time depending on tumour stage, microenvironment, TLS formation or applied therapies (including immunotherapy) remains to be explored.

## TLS are A Predictive Factor in The Response to Immunotherapy

Study of TLS in response to immunotherapy in sarcoma, melanoma and renal cell carcinoma patients showed strong associations between presence of TLS at the baseline and positive outcome of the ICB treatment (3–5). Whether TLS density increases in ICB-responding patients during the treatment is currently not clear. CD20 density (assessed by histology) was higher at the baseline for ICB responding patients and, crucially, was further increased after ICB therapy, while non-responding patients had low CD20 density before and after treatment (5). However, evaluation of TLS by histology did not show statistically significant increase in TLS density in ICB responders (compared to non-responders) although increased ratio of TLS to tumour was found for the former group (5). Studies with prospective validation with larger and more homogenous patient cohort will help to establish whether ICB therapy actively induces TLS formation, and if confirmed it will provide further strong evidence of active and beneficial role of TLS in immunotherapy.

Further evidence for positive role of TLS in immunotherapy comes from the analysis of sarcoma patients treated with anti-PD-1 blockade (3). Petitprez et al. divided sarcoma tumours into 5 different classes (based on the tumour microenvironment signature derived from RNA sequencing) and showed that tumours with high level of infiltration by immune cells (including B and T cell populations) carried strong prognostic value for improved patients’ survival prior to the ICB treatment. Interestingly, when response to anti-PD-1 immunotherapy was analysed, tumours with high immune cell infiltration lost their predictive value, unless they contained TLS. This study argues that what matters in response to immunotherapy is not only the presence of immune cells, but also their organisation into TLS.

## Humoral B Cell Responses in Immunotherapy

It is currently not clear whether B cells play an active role in anti-tumour responses e.g. *via* antibody-mediated mechanisms. Increased BCR diversity and expansion of memory B cells within the tumour was associated with the response to ICB in melanoma patients (5). However, no differences in the populations of GC-like B cells and plasma cells were found between responders and non-responders to ICB (5). Another study showed, however, expansion of CD69+ B cells with transcriptional signature resembling GC B cells in ICB-responding melanoma patients (4). More evidence is therefore needed to understand the extent to which the humoral functions of B cells contribute to immunotherapy response.

## Cross Talk Between B Cells and CD8 T Cells: An Exciting and Unexplored Avenue

One of the important unanswered questions surrounding response to ICB is whether there is a direct cross-talk between main subsets forming TLS (B cells) and effector cells directly involved in eliminating tumour response (e.g. CD8 T cells or natural killer cells).

There are several arguments speaking for a strong possibility of CD8 and B cell communication. The colocalization of B cells and CD8 T cells within the TLS is well established by numerous studies assessing TLS presence in several tumour entities [(37, 38, 43), reviewed in (34)]. Secondly, there is some evidence that CD8 T cells can actively recruit B cells to the tumours. Cabrita et al. (4) divided metastatic melanoma tumours in three groups (based on their T and B cell content), namely tumours with no T cells or B cells, containing T cells only or with both T and B cells present. The fact that no tumour group with B cells only was identified may suggest that T cells appear early at the tumour site and subsequently facilitate B cell recruitment. This is corroborated by several studies which identity CD8 T cells as a source of CXCL13 (47–49), a potent B cell chemoattractant. It is therefore reasonable to speculate that T cells possibly take active part in B cell recruitment and TLS formation, although that hypothesis remains to be vigorously tested.

**Figure 1:**
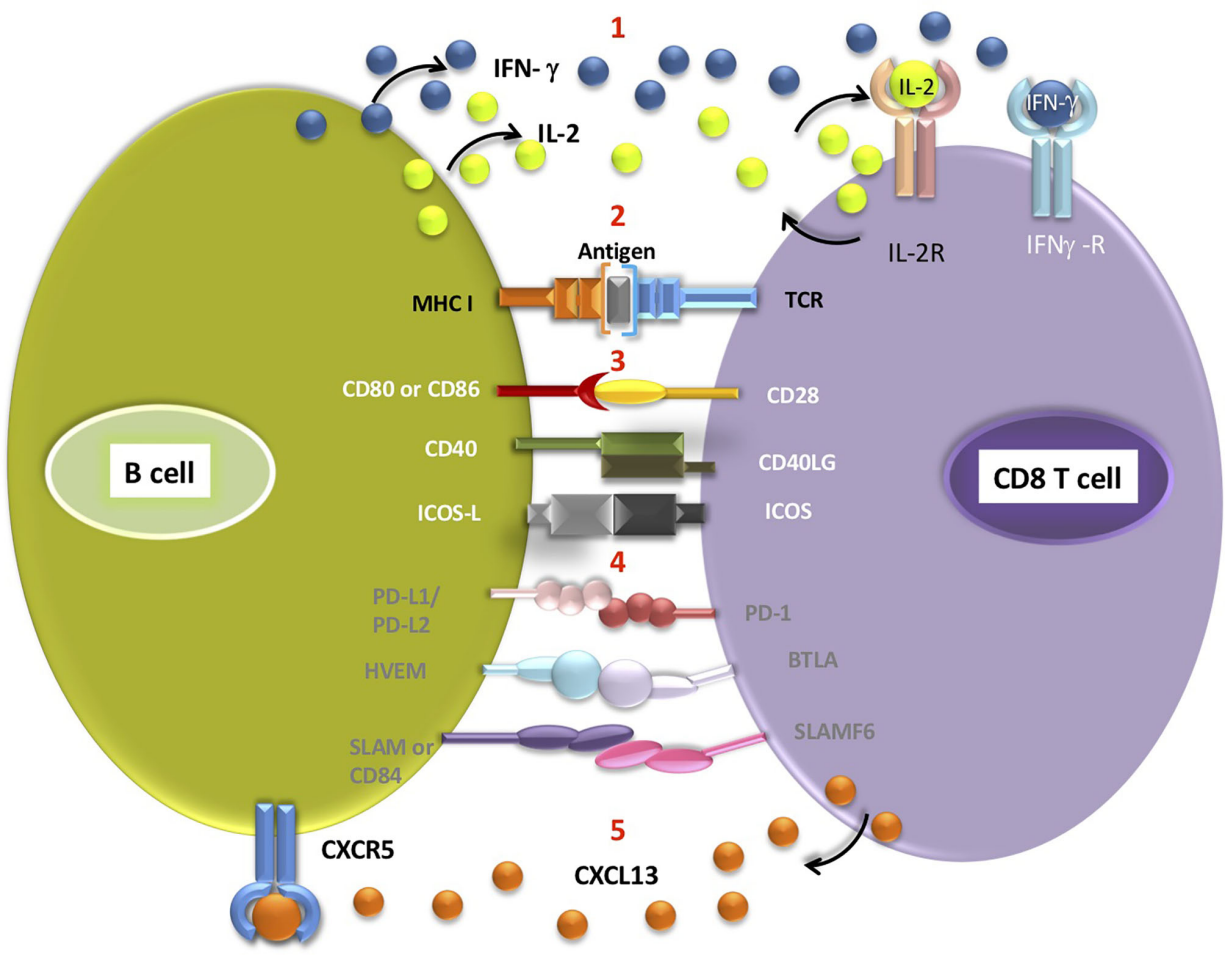
Interactions potentially occurring between the B cell and CD8 T cells located in the TLS (1). B cells secrete cytokines such as IFN-γ and IL-2, which can then bind to appropriate receptors on CD8 T cells (2). Antigen presentation on MHC class I molecule expressed by B cell triggers signalling from TCR receptor on T cell. Engagement of receptors on B cells and corresponding T cell receptors delivers co-stimulatory (3, white font) or co-inhibitory (4, grey font) signals to T cells (5). CD8 T cells secrete CXCL13, a B-cell chemoattractant which binds to CXCR5 receptors on B cells. Antigen cross-presentation by intratumoral B cells requires further experimental validation.

The potential advantages for CD8 T cells resulting from B cell recruitment are not fully understood. B cells exhibit a variety of functions, which can affect CD8 T cells directly or indirectly (**Figure 1**). Direct effects include antigen presentation, delivery of co-stimulatory or co-inhibitory receptors and cytokine secretion (38, 50), which can ultimately guide CD8 T cell activation, differentiation and effector function (as discussed above). An alternative explanation for TLS facilitating T cell-mediated ICB response is that it provides favourable niche for T cells to acquire their functional role, without direct role for B cell-mediated activation. As mentioned, other immune cell populations capable of antigen presentation are found within the TLS, such as mature CD208+ DCs (39, 51, 52). This mature DC subset was found to locate almost exclusively to TLS in lung cancer and is often used as a TLS marker (51). However, this phenomenon is limited to lung cancer and does not explain the supportive role of TLS in other tumour entities. Whether different antigen presenting cell populations support particular subsets of T cells, act at different stages of immune response to tumours or play interchangeable roles, remains to be seen. Additionally, B cells may support CD8 T cells indirectly *via* CD40-CD40L interactions with CD4+ T follicular helper cells, which would then provide enhanced help to effector T cells (53).

Furthermore, TLS creates microenvironment with a unique cytokine milieu (34). Apart from abundance of transcripts encoding cytokines involved in TLS generation (such as CXCL13, CCL19 and CCL21 (34, 50) many cytokines and chemokines involved in improving anti-tumour function and trafficking of T cells and DCs [eg. CXCR1, CCL20, IL-12, IFN-γ (38, 39)] and affecting other immune cells [e.g. macrophage chemoattractants CCL3, CCL4, CCL5 and neutrophil chemoattractant IL-8 (50)] are also found within the TLS.

Careful analysis of CD3 T cells (with both CD4 and CD8 populations included) revealed that T cells isolated from tumours with TLS have different transcription signature from T cells isolated from tumours without TLS, and this also differs to T cells isolated from TLS themselves (4). T cells isolated from B cell-rich tumours expressed more mRNA for *Tcf7* and *Tcf1* (involved in maintenance of stem-like T cell population), *Il7r* and *Sell* (encoding CD62L) as well as lower levels of transcripts for granzyme-encoding genes and *Ptpn22* (54), a negative regulator of TCR signalling (4). Additionally, analysis of CD8 T cells positioned within the TLS revealed elevated expression of activation markers compared to CD8 T cells placed outside TLS (5). This strongly supports the fact that there are significant differences in the gene and protein expression profile of T cells based on their location with respect to TLS. Interestingly, tumour-reactive, exhausted CD8+ T cell subset was found to locate predominantly in the TLS in lung cancer patients (47). Whether all CD8 T cells found within TLS are specific for tumour antigens and actively primed within the TLS remains an important and open question. Phenotypical, transcriptional and functional comparison of CD8 T cells positioned inside and outside TLS would reveal valuable insights about potential influence of TLS on cytotoxic effector T cells.

## Conclusions and Future Directions in TLS Investigation

Investigations of intratumoral T cells have paved the way in immunotherapy and their exploitation brought great clinical benefit to cancer patients. Currently the field is dynamically broadened to include other immune cells types and alternative immunotherapy approaches, including DC vaccinations (55), adoptive T cell and NK cell transfers (56) and agents targeting myeloid cell populations (57). Whether immunotherapeutic strategies can be tailored to target B cells remains to be determined, and the exact design of such therapies will depend on which B cell subset and function can be harnessed for the optimal clinical benefit. For example, it would be interesting to establish whether adoptive B cell transfer can lead to TLS induction (and recapitulate its complex spatial arrangement) to evoke durable immune response in cancer patients.

Future research will surely make most of the cutting-edge technologies, already employed for T cell-focused investigations, to characterise B cells and TLS. Although RNA sequencing [including single cell sequencing (58)] is an extremally useful tool for unbiased characterisation of intratumoral B cells, it does not contain information on spatial positioning of the cell within the tumour. On the other hand, multiplex imaging technologies are getting advanced at great speed to enable visualisation of up to 50-60 markers from a single tissue slide (59, 60). This is a very useful approach for thorough analysis of TME, and especially immune compartment, with the limitations of its biased approach (as analysis is restricted to antibodies available for imaging). Spatial sequencing, with provides information on transcriptome composition together with tissue coordinate of each cell, is a perfect tool for TLS-based investigation. However, in this case transcriptome analysis is derived from a cluster of cells rather than a single cell [although resolution of this technology is increasing rapidly (61)], which can lead to difficulties in deciphering gene expression profiles from adjacent T and B cells. Therefore, combination of unbiased and targeted experimental approach (62, 63) is an exciting direction for deciphering role of TLS in cancer immunotherapy.

Our appreciation of TLS and their active role in immunotherapy is continuously growing, but the phenomenon is still far from being completely understood. It would be of great importance to the scientific community to address the question of ICB treatment on phenotype and function of B cell populations. Studies addressing functional connections to CD8 T cells in ICB would optimally guide the treatment strategies targeting selected aspects of TLS and B cell biology. Identification of mechanistic insights will provide an exciting and immediate avenue for translation of B cell-based immunotherapies into clinics, complementary to existing T cell-centric strategies.

## Author Contributions

MT and AZ wrote the manuscript. All authors contributed to the article and approved the submitted version.

## Funding

MT is supported by University of Basel Research Grant and Cancer League Basel. AZ is supported by grants from the Swiss National Science Foundation (320030_188576/1; CRSII5_170929).

## Conflict of Interest

The authors declare that the research was conducted in the absence of any commercial or financial relationships that could be construed as a potential conflict of interest.
